# A Role for Gene-Environment Interactions in Autism Spectrum Disorder Is Supported by Variants in Genes Regulating the Effects of Exposure to Xenobiotics

**DOI:** 10.3389/fnins.2022.862315

**Published:** 2022-05-19

**Authors:** João Xavier Santos, Célia Rasga, Ana Rita Marques, Hugo Martiniano, Muhammad Asif, Joana Vilela, Guiomar Oliveira, Lisete Sousa, Ana Nunes, Astrid M. Vicente

**Affiliations:** ^1^Departamento de Promoção da Saúde e Doenças Não Transmissíveis, Instituto Nacional de Saúde Doutor Ricardo Jorge, Lisbon, Portugal; ^2^BioISI–Biosystems and Integrative Sciences Institute, Faculty of Sciences, University of Lisboa, Lisbon, Portugal; ^3^Unidade de Neurodesenvolvimento e Autismo, Serviço do Centro de Desenvolvimento da Criança, Centro de Investigação e Formação Clínica, Hospital Pediátrico, Centro Hospitalar e Universitário de Coimbra, Coimbra, Portugal; ^4^Faculty of Medicine, University Clinic of Pediatrics and Coimbra Institute for Biomedical Imaging and Translational Research, University of Coimbra, Coimbra, Portugal; ^5^Departamento de Estatística e Investigação Operacional e Centro de Estatística e Aplicações, Faculdade de Ciências, Universidade de Lisboa, Lisbon, Portugal; ^6^Departamento de Física, Faculdade de Ciências, Universidade de Lisboa, Lisbon, Portugal

**Keywords:** autism spectrum disorder, blood-brain barrier, detoxification, gene-environment interactions, placenta, xenobiotics exposure

## Abstract

Heritability estimates support the contribution of genetics and the environment to the etiology of Autism Spectrum Disorder (ASD), but a role for gene-environment interactions is insufficiently explored. Genes involved in detoxification pathways and physiological permeability barriers (e.g., blood-brain barrier, placenta and respiratory airways), which regulate the effects of exposure to xenobiotics during early stages of neurodevelopment when the immature brain is extremely vulnerable, may be particularly relevant in this context. Our objective was to identify genes involved in the regulation of xenobiotic detoxification or the function of physiological barriers (the XenoReg genes) presenting predicted damaging variants in subjects with ASD, and to understand their interaction patterns with ubiquitous xenobiotics previously implicated in this disorder. We defined a panel of 519 XenoReg genes through literature review and database queries. Large ASD datasets were inspected for *in silico* predicted damaging Single Nucleotide Variants (SNVs) (*N* = 2,674 subjects) or Copy Number Variants (CNVs) (*N* = 3,570 subjects) in XenoReg genes. We queried the Comparative Toxicogenomics Database (CTD) to identify interaction pairs between XenoReg genes and xenobiotics. The interrogation of ASD datasets for variants in the XenoReg gene panel identified 77 genes with high evidence for a role in ASD, according to pre-specified prioritization criteria. These include 47 genes encoding detoxification enzymes and 30 genes encoding proteins involved in physiological barrier function, among which 15 are previous reported candidates for ASD. The CTD query revealed 397 gene-environment interaction pairs between these XenoReg genes and 80% (48/60) of the analyzed xenobiotics. The top interacting genes and xenobiotics were, respectively, *CYP1A2*, *ABCB1*, *ABCG2*, *GSTM1*, and *CYP2D6* and benzo-(a)-pyrene, valproic acid, bisphenol A, particulate matter, methylmercury, and perfluorinated compounds. Individuals carrying predicted damaging variants in high evidence XenoReg genes are likely to have less efficient detoxification systems or impaired physiological barriers. They can therefore be particularly susceptible to early life exposure to ubiquitous xenobiotics, which elicit neuropathological mechanisms in the immature brain, such as epigenetic changes, oxidative stress, neuroinflammation, hypoxic damage, and endocrine disruption. As exposure to environmental factors may be mitigated for individuals with risk variants, this work provides new perspectives to personalized prevention and health management policies for ASD.

## Introduction

Autism Spectrum Disorder (ASD) is an early onset neurodevelopmental disorder characterized by deficits in social communication and interaction, and repetitive and stereotyped interests and behaviors (American Psychiatric Association, 2013). This disorder is phenotypically very heterogeneous, and often occurs with comorbidities such as intellectual disability, speech and language impairments and attention-deficit/hyperactivity disorder (Lord et al., 2020). The etiology of ASD is unclear, but a prevalent hypothesis is that of a multifactorial origin, with genetic and environmental risk factors interacting cumulatively toward a threshold for disease onset (Persico and Merelli, 2014). Environmental factors might interact with the genetic background of an individual, either by triggering or modulating the phenotypic expression of genetic risk factors or by exerting additive or synergistic effects, originating a variable spectrum of susceptibility to environmental factors (Persico and Merelli, 2014). This concept is supported by ASD heritability estimates of 50–83% (Sandin et al., 2017; Bai et al., 2019; Pettersson et al., 2019), leaving ample space for a role of gene-environment interactions in its etiology. Over the past decade, genomic studies have identified many rare, *de novo* or inherited, Copy Number Variants (CNVs) (Pinto et al., 2010, 2014; Sanders et al., 2011) and/or gene-disrupting Single Nucleotide Variants (SNVs) associated with the pathology (Iossifov et al., 2014; Feliciano et al., 2019). A genetic etiology may be found in up to 40% of ASD cases (Schaefer et al., 2013; Genovese and Butler, 2020), including known genetic syndromes, metabolic and mitochondrial dysfunctions, chromosomal deletions or duplications and genetic variants in hundreds of genes detected by exome and genome sequencing (Bourgeron, 2015; Chen et al., 2015). However, a significant number of patients still remain idiopathic, suggesting a highly complex genetic architecture compounded by environmental influences.

Early stages of development are a recognized window of susceptibility to environmental stimuli that can have detrimental effects, potentially modulating the neuropathological events that lead to ASD onset (Pinson et al., 2016). Recent studies have suggested that prenatal to early postnatal (i.e., from preconception to the 2nd year of life) exposure to ubiquitous xenobiotics may constitute a risk factor for ASD (reviewed in Santos et al., 2021). For instance, early exposure to air pollutants, such as nitrogen dioxide (NO_2_), ozone (O_3_), particulate matter (PM), sulfur dioxide (SO_2_), and polycyclic aromatic hydrocarbons (PAHs), has been consistently associated with ASD risk (Rossignol et al., 2014; Modabbernia et al., 2017; Ritz et al., 2018; Santos et al., 2021). There is also evidence that exposure to persistent organic pollutants (POPs) [polybrominated diphenyl ethers (PBDEs), polychlorinated biphenyls (PCBs), and perfluorinated compounds (PFCs)] and to non-persistent organic pollutants (non-POPs) [bisphenol A (BPA) and phthalates] is a risk factor for ASD (Rossignol et al., 2014; Modabbernia et al., 2017; Santos et al., 2021). Such toxins are present in everyday household and industrial products and food. POPs are resistant to biodegradation, which increases their risk of bioaccumulation. Conversely, BPA and phthalates are rapidly metabolized but, due to their application in plastic-based consumer goods such as canned or packaged food, water bottles and toys, exposure to these compounds is virtually continuous and ubiquitous. Other environmental toxins previously implicated in ASD include heavy metals (lead, manganese, and mercury) (Modabbernia et al., 2017; Santos et al., 2021) and pesticides (Rossignol et al., 2014; Modabbernia et al., 2017; Santos et al., 2021). Maternal pregnancy intake of teratogenic drugs, such as valproic acid (Christensen et al., 2013; Modabbernia et al., 2017), thalidomide (Stromland et al., 1994), and misoprostol (Bandim et al., 2003), as well as antidepressants, particularly selective serotonin reuptake inhibitors (SSRIs) (Modabbernia et al., 2017; Morales et al., 2018), has also been reported to increase ASD risk in the offspring. Most of these xenobiotics are known to have neurotoxic properties (Landrigan, 2010) and many are recognized endocrine-disrupting chemicals (EDCs) (Schug et al., 2015). Finally, insufficient gestational or postnatal levels of nutritional factors, including folic acid and vitamin D (Modabbernia et al., 2017), have been associated with increased risk of developing ASD.

Crucial in regulating the harmful effects of these xenobiotics to the developing organism are physiological detoxification processes and physiological permeability barriers. Detoxification pathways involve series of enzymatic reactions that act to detoxify xenobiotics and remove them, or their metabolites, from cells. These are mediated by large families of molecules, including cytochromes P450 (CYPs), UDP-glucuronosyltransferases (UGTs), glutathione S-transferases (GSTs), and others. Most of these enzymes are encoded by highly polymorphic genes, with variants affecting the metabolizer status of their carriers (Santos et al., 2018).

Physiological permeability barriers include the placenta, the blood-brain barrier (BBB) and the motile cilia of the human airway epithelia, which limit the exposure of the organism to chemicals. The placenta establishes an interface between the mother and the developing fetus that regulates the transfer of nutrients and waste products between maternal and fetal blood. While the placenta is the first fetal line of defense against direct contact with xenobiotics during pregnancy, the BBB has been shown to be functional as early as at 8 weeks of gestation, and by the 12th week there is expression of tight junction proteins (Goasdoué et al., 2017; Kadry et al., 2020). In the BBB, the tight junctions formed between the endothelial cells confer a semi-permeability to various neurotoxins, and therefore form a barrier to the free movement of molecules from the early stages of brain development (Kadry et al., 2020). The respiratory epithelium serves as a barrier to xenobiotics through the action of mucociliary clearance carried by the cilia. The regulation of the selective permeability of physiological barriers is particularly relevant for a healthy neurodevelopment, when the organism is more vulnerable to exogenous influences. However, since these structures are semi-permeable, they are not impenetrable to all toxins. For instance, while the placenta prevents the flux of neurotoxins from maternal to fetal blood, its barrier capacity is limited and variable concentrations of xenobiotics, such as BPA (Tang et al., 2020), phthalates (Tang et al., 2020), pesticides (Acosta-Maldonado et al., 2009), and heavy metals (Gundacker and Hengstschläger, 2012) have been detected in fetal bloodstream. Once in the fetus, these toxins may also cross the tight junctions of the BBB and contact directly with the immature brain (Agúndez et al., 2014, reviewed by Santos et al., 2021). Transporters, such as the ATP-binding cassette (ABCs) pumps and Solute Carriers (SLCs) are particularly important at the BBB and placenta, as they control the transmembrane uptake and efflux of substances across these barriers.

In this study we explore the hypothesis that individuals carrying functional variants in genes that regulate detoxification processes or the permeability of physiological barriers to xenobiotics (from here on termed XenoReg genes) may be more vulnerable to the harmful effects of early life exposure to ubiquitous xenobiotics, and therefore have an increased ASD risk. In large datasets of individuals with ASD, we sought to identify predicted damaging variants in XenoReg genes, and explore their interactions with xenobiotics previously associated with this condition. Beyond providing new insights for ASD etiology, the discovery of relevant gene-environment interactions opens novel perspectives for ASD prevention, given the possibility of mitigating exposure when a genetic vulnerability is identified.

## Materials and Methods

**FIGURE 1:**
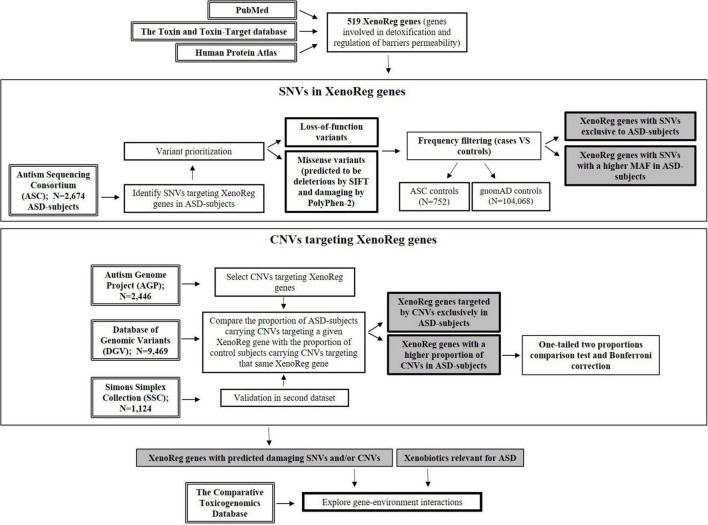
Flowchart resuming the workflow of this study. Genes involved in detoxification and regulation of barriers permeability—XenoReg genes—were identified through PubMed and databases (The Human Protein Atlas and the Toxin and Toxin-Target Database). Large population datasets were interrogated for the presence of predicted damaging SNVs and CNVs in XenoReg genes, in individuals with ASD. The Comparative Toxicogenomics Database was queried to identify interactions between high evidence XenoReg genes and xenobiotics previously implicated in the disorder. ASD, Autism Spectrum Disorder; CNVs, Copy Number Variants; MAF, Minor Allele Frequency; SNVs, Single Nucleotide Variants; XenoReg genes, genes involved in regulation of detoxification and physiological barriers permeability to xenobiotics.

For a flowchart summarizing the complete workflow used in the current study (see Figure 1).

### Defining a Panel of Genes Involved in Detoxification and Regulation of Barriers Permeability to Xenobiotics (XenoReg Genes Panel)

To define a panel of XenoReg genes we performed a literature review, by querying PubMed (RRID: SCR_004846) (Figure 1) with the following terms: “detoxification,” “placenta,” “blood-brain barrier,” and “respiratory cilia.” We limited our query output to English-language article reviews published up to 2017. We also interrogated publicly available databases, namely The Human Protein Atlas (HPA)^1^ (Uhlén et al., 2015) (RRID: SCR_006710) and the Toxin and Toxin-Target Database (T3DB) (Lim et al., 2010; Wishart et al., 2015) (RRID: SCR_002672). The Human Protein Atlas contains protein expression data derived from immunohistochemical staining of specific cell populations in human tissues and organs, including the placenta, and was thus used for the identification of genes highly expressed in this structure. T3DB provides mechanisms of toxicity and target proteins for a wide variety of toxins, allowing us to select genes encoding proteins that interact with xenobiotics.

### Population Datasets

To support a role of gene-environment interactions in ASD, we inspected large datasets of ASD individuals for variants in XenoReg genes, including SNVs and CNVs (Figure 1).

For SNV discovery we used exome-sequencing data from 3,426 subjects (2,674 ASD cases and 752 unrelated ancestry-matched controls) from the Autism Sequencing Consortium (ASC) (Supplementary Table 1). This international consortium aimed to use high-throughput sequencing techniques to identify genetic risk factors for ASD (Buxbaum et al., 2012). ASC data was available through dbGaP portal (accession code: phs000298.v4.p3). Exome-sequencing data from 104,068 subjects not ascertained for having a neurological condition in case/control studies, deposited in the Genome Aggregation Database (gnomAD) (RRID: SCR_014964), was used to estimate variant frequencies in the general population (Karczewski et al., 2020).

For CNV identification we analyzed genetic data from the Autism Genome Project (AGP) consortium (*N* = 2,446) (accession code: phs000267.v5.p2) (Pinto et al., 2010, 2014; Supplementary Table 1) and from the Simons Simplex Collection (SSC) (*N* = 1,124) (Sanders et al., 2011) datasets, amounting to a total of 3,570 subjects with genome-wide CNV data. The AGP is a large-scale, international research consortium designed to identify autism candidate genes (Hu-Lince et al., 2005), while the SSC is a resource from the Simons Foundation for Autism Research (SFARI) (Fischbach and Lord, 2010) (RRID: SCR_004261). CNV data from two cohorts of subjects without clinical history of neuropsychiatric disease (NPD) (*N* = 9,649) was used to estimate CNV frequencies in the general population (Shaikh et al., 2009; Cooper et al., 2011; Supplementary Table 1). CNV data from these control datasets are publicly available through the Database of Genomic Variants (DGV) (MacDonald et al., 2014; RRID: SCR_007000; Supplementary Table 1). All these populations were genotyped using Illumina arrays.

ASC, AGP, and SSC datasets obtained genetic data from family trios. Maternal genetic data was available for 1,702 (63.6%) subjects from ASC, 2,241 (91.6%) subjects from AGP, and 778 (70.1%) subjects from SSC. In these subjects we assessed the transmission pattern of selected SNVs and CNVs in XenoReg genes encoding detoxification enzymes. Because some subjects may be common between these datasets, we analyzed each dataset separately.

The gold standard Autism Diagnostic Interview-Revised (ADI-R) (Lord et al., 1994) and/or the Autism Diagnostic Observation Schedule (ADOS) tools (Lord et al., 2000) were applied for clinical assessment and ASD diagnosis in the ASC, AGP, and SSC datasets (Pinto et al., 2010, 2014; Sanders et al., 2011; Buxbaum et al., 2012).

### Single Nucleotide Variant Analysis and Prioritization

Quality control of exome-sequencing data from ASC was done by filtering out samples with minimum depth filter < 8 and genotype quality ≤ 20 and by excluding variants with missingness > 10%. Very common variants in the general population, with a Minor Allele Frequency (MAF) > 5% on gnomAD, were not considered. Variant functional annotation was performed using the Variant Effect Predictor (VEP, version 86) (RRID: SCR_007931) tool from Ensembl (McLaren et al., 2016), with human genome built 37 (GRCh37/hg19) as reference, allowing us to assess the functional impact predictions attributed to non-synonymous (missense and loss of start codon) mutations by SIFT (Sim et al., 2012) (RRID: SCR_012813) and PolyPhen-2 (Adzhubei et al., 2010) (RRID: SCR_013189) *in silico* tools.

For variant prioritization, we only selected loss-of-function (LoF) and missense variants, predicted as having high and moderate impact by VEP, respectively. LoF variants include frameshift mutations, loss of start or stop codons, gain of a stop codon and mutations in splice donor and acceptor sites. For missense impact variants, we only selected those predicted as deleterious by SIFT and probably or possibly damaging by PolyPhen-2. We also examined the frequency of the selected variants in cases and controls, and considered only SNVs exclusively present in cases or with a higher MAF in cases when compared to ASC controls (MAF_cases_/MAF_ASC_controls_ > 1.5). Most of the SNVs exclusive in cases were found in only 1 or 2 affected subjects, and might not be identified in ASC controls because of the smaller size of this population. To overcome this issue, we examined the MAF of the variants that were more frequent or exclusive to ASC cases, when compared to ASC controls, in exome-sequencing data from 104,068 subjects without neurological disorder from gnomAD. The gnomAD public dataset aggregates and harmonizes exome and genome sequencing data from large-scale projects, using processing procedures to ensure consistency across projects that include hundreds of thousands of subjects. Using this set of controls we identified the XenoReg genes with predicted damaging SNVs exclusively present or more frequent in ASD-subjects when compared to controls without history of neurological disorder (Figure 1).

### Copy Number Variant Analysis

CNVs targeting XenoReg genes were obtained from the AGP, SSC, and DGV datasets (Supplementary Table 1). Genotyping and CNV calling for the AGP participants has been previously described by Pinto et al. (2010). High-confidence rare CNVs, predicted by at least two of three calling algorithms or experimentally validated by real time quantitative PCR (Pinto et al., 2014), and with a frequency below 1% in the AGP dataset, were analyzed. For the SSC dataset, variants were previously defined as rare when up to 50% of their sequence overlapped with regions present at less than 1% frequency in DGV controls (MacDonald et al., 2014).

We analyzed the frequencies of CNVs targeting each of the XenoReg genes in ASD cases vs. subjects without clinical history of NPD. For this, we applied a two proportions comparison test, which consists on a one-tailed test to establish whether the proportion of ASD-subjects carrying CNVs targeting a given gene (*p*_ASD cases_) is higher than the proportion of non-NPD subjects carrying CNVs targeting that same gene (*p*_non–NPD subjects_), where *p* stands for proportion. The null hypothesis (H0) is defined by *p*_ASD cases_ ≤ *p*_non–NPD subjects_, while the alternative hypothesis (H1) is given by *p*_ASD cases_ > *p*_non–NPD subjects_. Bonferroni correction for multiple testing was applied, with significance at α = 0.05. Moreover, we searched for XenoReg genes exclusively targeted by CNVs in ASD patients. Statistical analyses were performed using the open source software environment R.

### Characterization of High Evidence XenoReg Genes

A brief characterization of prioritized high evidence XenoReg genes was carried out. Gene symbol and name are in accordance with HUGO Gene Nomenclature Committee (HGNC) guidelines (RRID: SCR_002827). GeneCards (RRID: SCR_002773) was used to find the cytogenetic location of the genes and function of the encoded proteins. The HPA was used to establish the tissue expression profile of the selected genes. The Human Gene Module from SFARI, a manually curated list of candidate genes for ASD originating from publications in peer-reviewed journals (Abrahams et al., 2013), was used to identify genes previously associated with the disorder. To complement this, PubMed was also interrogated, using combinations between the symbols of the genes and the term “ASD” for association and expression profiling studies linking these genes with ASD.

### Interactions Between XenoReg Genes and Xenobiotics Potentially Relevant for Autism Spectrum Disorder

To explore interactions between xenobiotics previously associated with ASD risk and XenoReg genes with predicted damaging variants in ASD subjects, we resorted to the Comparative Toxicogenomics Database (CTD) (RRID: SCR_006530) (Figure 1). CTD is a manually curated platform that provides information about interactions between chemicals and gene products (Davis et al., 2020), and lists all published references that support each interaction. As of March, 2021, 2,267,845 chemical-gene interactions between 51,993 unique genes and 13,844 unique chemicals in 611 organisms were recorded by the CTD.

We uploaded the HGNC gene symbols of high evidence XenoReg genes to the CTD query interface. The output files were manually interrogated for the presence of the MeSH IDs (RRID: SCR_004750) corresponding to each chemical. Only interactions observed in *Homo sapiens* were considered. We surveyed 60 individual chemicals previously associated with ASD, as listed in Supplementary Table 2 (Santos et al., 2021). These chemicals are organized in seven major groups: Air Pollutants, Toxic Heavy Metals, Non-Persistent Organic Pollutants, Persistent Organic Pollutants, Pesticides, Clinical Drugs, and Nutritional Factors (Santos et al., 2021). Among the selected toxic heavy metals were elemental mercury and its derivative methylmercury (MeHg), which have been shown to impact neurodevelopment (Debes et al., 2006; Yoshimasu et al., 2014; Modabbernia et al., 2017) but not ethylmercury (etHg).

## Results

### Genes Involved in Detoxification and Regulation of Barriers Permeability to Xenobiotics (XenoReg Genes)

A literature review and public database queries (Human Protein Atlas and T3DB), identified 519 genes involved in detoxification processes or in the regulation of integrity or permeability of physiological barriers to xenobiotics (XenoReg genes) (Table 1). The detoxification genes encoded enzymes involved in Phase I (e.g., CYPs) and Phase II (e.g., UGTs, GSTs, sulfatases, and sulfotransferases) metabolic pathways, which consist in a series of reactions that increase hydrophilicity of xenobiotics to facilitate disposition, maintaining cellular homeostasis. Genes expressed in physiological barriers encoded transporters and receptors (e.g., ATP-binding cassette transporters and solute carriers) that regulate the permeability of these structures, as well as hormones and proteins responsible for their integrity and morphogenesis. For a full list of these genes refer to Supplementary Table 3.

**TABLE 1:** Main classes of genes involved in detoxification and permeability regulation of the BBB, placenta or respiratory cilia (XenoReg genes).

Genes	Number of genes
**Detoxification genes**	**297**
Cytochromes P450 (CYPs)	57
Glutathione peroxidases	7
Glutathione S-transferases (GSTs)	21
Methyltransferases	9
Sulfatases	17
Sulfotransferases	32
UDP-glucuronosyltransferases (UGTs)	19
Other genes involved in detoxification	135
**Blood-brain barrier genes**	**83**
ATP-binding cassette transporters (ABCs)	7
Solute carriers (SLCs)	29
Na^+^/K^+^ ATPase subunits	8
Brain endothelial junctional complex proteins	27
Other genes highly expressed at the BBB	12
**Placenta genes**	**112**
Pregnancy-specific glycoproteins	10
Hormones expressed at the placenta	12
Placenta receptors and transporters	17
Genes related to placenta morphogenesis	22
Other genes highly expressed at the placenta	45
**Respiratory cilia genes**	**27**

*Proteins encoded by these genes may overlap in their roles [e.g., while detoxification occurs mainly in the liver, some CYPs and UGTs are also expressed at the barrier structures (Syme et al., 2004; Agúndez et al., 2014)].*

### Discovery of Predicted Damaging Single Nucleotide Variants in XenoReg Genes in Autism Spectrum Disorder Subjects

**FIGURE 2:**
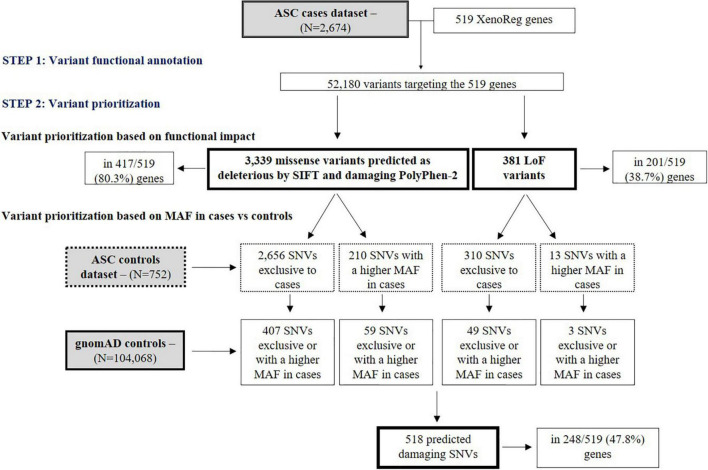
Flowchart of main results regarding SNV analyses. Shown are the numbers of LoF and missense (predicted as deleterious by SIFT and damaging by PolyPhen-2) SNVs in XenoReg genes, in ASD cases from ASC when compared to controls from ASC and gnomAD. The functional impact of variants present in ASD cases was predicted by VEP and only LoF and missense SNVs predicted to be deleterious by SIFT and damaging PolyPhen-2 were considered. The MAF of these variants was then compared with that from ASC controls and gnomAD subjects not ascertained for having a neurological disorder. ASC, Autism Sequencing Consortium; ASD, Autism Spectrum Disorder; gnomAD, Genome Aggregation Database; MAF, Minor Allele Frequency.

ASC exome-sequencing data from 2,674 subjects with ASD was analyzed for the presence of SNVs in the 519 XenoReg genes. In these subjects, we identified 3,339 missense and 381 LoF variants (total *N* = 3,720) predicted to be damaging by SIFT and PolyPhen-2 tools, in 417 and 201 XenoReg genes, respectively (Figure 2). Overall, 80.9% of the 519 XenoReg genes had at least one missense or LoF variant. We further observed that 85.7% (3,189/3,720) of the variants predicted to be damaging, including 2,866 missense SNVs and 323 LoF SNVs, were either only present in cases or more frequent in cases when compared to ASC controls (Figure 2). To overcome the issues raised by the smaller size of the ASC control dataset, we examined the MAF of these 3,189 variants in 104,068 subjects without neurological disorder from gnomAD (Figure 2). We identified 518 unique variants exclusive to ASD cases or with a higher MAF in cases when compared to gnomAD controls. These 518 SNVs were found in 47.8% (248/519) of the XenoReg genes (Figure 2) listed in Supplementary Table 4.

### Discovery of Copy Number Variants in XenoReg Genes in Autism Spectrum Disorder Subjects

We sought to identify CNVs targeting XenoReg genes in ASD-subjects from the AGP (*N* = 2,446) and SSC (*N* = 1,124) datasets.

In the AGP dataset, 31 (6.0%) of the 519 genes in the XenoReg gene list were targeted by CNVs exclusively in ASD-subjects and in none of 9,649 non-NPD controls from DGV (Table 2). CNVs in eight (*STS, CYP2D6, ARSF, CLDN3, GUSB, CYP2R1, SLC3A2, and SULT2B1*) of these 31 genes were also found in the SSC dataset (Table 2). Overall, 46 XenoReg genes were targeted by CNVs exclusively in ASD-subjects (8 genes found in both AGP and SSC datasets, 23 genes only in AGP dataset, and 15 genes only in SSC dataset) (Table 2).

**TABLE 2:** Frequencies observed for XenoReg genes targeted by CNVs exclusively in individuals with ASD from the AGP and/or SSC datasets, when compared to controls from the DGV dataset.

Genes exclusively targeted by CNVs in both AGP (*N* = 2,446) and SSC (*N* = 1,124) subjects			Genes exclusively targeted by CNVs only in AGP subjects (*N* = 2,446)		Genes exclusively targeted by CNVs only in SSC subjects (*N* = 1,124)	
Gene	AGP n (%)	SSC n (%)	Gene	AGP n (%)	Gene	SSC n (%)
*STS*	12 (0.491)	7 (0.623)	*ARSE*	3 (0.123)	*SGF29*	4 (0.356)
*CYP2D6*	9 (0.368)	5 (0.445)	*ARSH*	3 (0.123)	*CHST14*	3 (0.267)
*ARSF*	5 (0.204)	1 (0.089)	*SLC16A1*	3 (0.123)	*ADSL*	2 (0.178)
*GUSB*	3 (0.123)	1 (0.089)	*TRIM64B*	3 (0.123)	*CYP4F22*	2 (0.178)
*CLDN3*	1 (0.041)	4 (0.356)	*ABCG2*	2 (0.082)	*HSD17B1*	2 (0.178)
*CYP2R1*	1 (0.041)	1 (0.089)	*AKR7A3*	2 (0.082)	*CHST4*	1 (0.089)
*SLC3A2*	1 (0.041)	1 (0.089)	*ALDH3A2*	2 (0.082)	*CYP4A11*	1 (0.089)
*SULT2B1*	1 (0.041)	1 (0.089)	*CHST12*	2 (0.082)	*CYP4F2*	1 (0.089)
			*XAGE3*	2 (0.082)	*CYP4F8*	1 (0.089)
			*ARSG*	1 (0.041)	*DNMT3B*	1 (0.089)
			*CES3*	1 (0.041)	*JAM2*	1 (0.089)
			*CYP11B2*	1 (0.041)	*PTGES*	1 (0.089)
			*CYP7A1*	1 (0.041)	*PTGS1*	1 (0.089)
			*FMO3*	1 (0.041)	*SLC22A5*	1 (0.089)
			*GPX2*	1 (0.041)	*SYN1*	1 (0.089)
			*IL1RL1*	1 (0.041)		
			*KIFC1*	1 (0.041)		
			*NOTUM*	1 (0.041)		
			*PTGES3*	1 (0.041)		
			*SLC25A20*	1 (0.041)		
			*SUOX*	1 (0.041)		
			*UGT1A1*	1 (0.041)		
			*UGT2A3*	1 (0.041)		

*AGP, Autism Genome Project; CNVs, Copy Number Variants; SSC, Simons Simplex Collection.*

For XenoReg genes targeted by CNVs in both ASD and non-NPD control subjects from DGV we performed a one-tailed two proportions comparison test. This test showed that 11 genes had a higher proportion of CNVs in AGP ASD-subjects when compared to controls, after Bonferroni correction for multiple testing (*p* < 0.05) (Table 3). From these 11 genes, *CHST5* and *MAGEA8* were also found to have a statistically significant higher proportion of CNVs in SSC subjects when compared to non-NPD controls (Table 3) (*p* < 0.05). There were no XenoReg genes with a higher proportion of CNVs in the SSC dataset but not in AGP dataset, when compared to non-NPD controls.

**TABLE 3:** Summary statistics for the XenoReg genes with a higher proportion of CNVs in individuals with ASD from AGP and/or SSC datasets when compared to controls from the DGV dataset.

	AGP dataset (*N* = 2,446)				SSC dataset (*N* = 1,124)				Control dataset (*N* = 9,649)
	AGP n (%)	Test statistic	*p*-value	Adjusted *p*-value	SSC n (%)	Test statistic	*p*-value	Adjusted *p*-value	DGV n (%)
*CYP21A2*	67 (2.739)	5.08	1.96 × 10^–07^	2.78 × 10^–05^	23 (2.046)	2.44	7.32 × 10^–03^	7.97 × 10^–01^	95 (0.985)
***CHST5***	33 (1.349)	4.23	1.21 × 10^–05^	1.71 × 10^–03^	23 (2.046)	4.02	3.10 × 10^–05^	3.37 × 10^–03^	32 (0.332)
*ABCB1*	20 (0.818)	4.17	1.56 × 10^–05^	2.22 × 10^–03^	2 (0.178)	0.99	1.62 × 10^–01^	1	5 (0.052)
*GSTM1*	20 (0.818)	4.17	1.56 × 10^–05^	2.22 × 10^–03^	2 (0.178)	0.99	1.62 × 10^–01^	1	5 (0.052)
*CYP4X1*	17 (0.695)	4.00	3.29 × 10^–05^	4.67 × 10^–03^	7 (0.623)	2.60	5.31 × 10^–03^	5.78 × 10^–01^	2 (0.021)
***MAGEA8***	16 (0.654)	3.94	4.17 × 10^–05^	5.93 × 10^–03^	15 (1.335)	3.87	5.86 × 10^–05^	6.33 × 10^–03^	1 (0.010)
*CSH1*	19 (0.777)	3.92	4.54 × 10^–05^	6.45 × 10^–03^	9 (0.801)	2.72	3.28 × 10^–03^	3.57 × 10^–01^	7 (0.073)
*GSTT2*	16 (0.654)	3.66	1.30 × 10^–04^	1.84 × 10^–02^	1 (0.089)	0.40	3.43 × 10^–01^	1	5 (0.052)
*UGT1A8*	14 (0.572	3.60	1.63 × 10^–04^	2.31 × 10^–02^	3 (0.267)	1.59	5.59 × 10^–02^	1	2 (0.021)
*UGT2B10*	14 (0.572)	3.60	1.63 × 10^–04^	2.31 × 10^–02^	0 (0)	n/a	n/a	n/a	2 (0.021)
*UGT1A10*	13 (0.531)	3.46	2.78 × 10^–04^	3.95 × 10^–02^	3 (0.267)	1.59	5.59 × 10^–02^	1	2 (0.021)

*A conservative Bonferroni correction for multiple comparisons was applied, with statistical significance at p < 0.05. The table shows genes with a higher proportion of CNVs in both the AGP and SSC datasets (in bold and underlined), or with a higher proportion only in the AGP dataset (not in bold). Supplementary Table 5 shows the test results for the XenoReg genes that did not reach statistical significance. AGP, Autism Genome Project; CNVs, Copy Number Variants; SSC, Simons Simplex Collection.*

### XenoReg Genes With High Evidence for a Role in Autism Spectrum Disorder

Overall, in the ASD datasets we found that 281 genes of the XenoReg panel carried predicted damaging SNVs (*N* = 248) and/or CNVs (*N* = 57), as indicated by (1) their *in silico* predicted functional impact, and exclusive identification or higher MAF in ASD cases (for SNVs), (2) exclusive identification or higher proportion in ASD cases vs. non-NPD controls (for CNVs). Of these 281 genes, 24 carried both SNVs and CNVs in ASD subjects.

Of the 281 XenoReg genes with predicted damaging SNVs and CNVs, we prioritize 77 candidates with a high evidence for a role in ASD (Table 4), based on three criteria: (1) genes with both risk SNVs and CNVs (*N* = 24) in cases; (2) genes that had 5 or more unique risk SNVs (*N* = 20), but no CNVs, in cases; (3) genes targeted by CNVs exclusively in cases (*N* = 26), or showing a higher proportion of CNVs in cases (*N* = 7), but with no SNVs. Predicted damaging SNVs in these 77 high evidence XenoReg genes were identified in 15.6% (417/2,674) ASD subjects from ASC, while predicted damaging CNVs were found in 11.5% (282/2,446) and 6.9% (77/1,124) of ASD-subjects from AGP and SSC, respectively (Supplementary Table 6).

**TABLE 4:** XenoReg genes with a high evidence for a role in ASD.

Selection criteria	Gene category	Previous evidence for a role in ASD	No previous evidence for a role in ASD
XenoReg genes with predicted damaging SNVs and CNVs in cases (*n* = 24)	DET	*CHST5, CYP2R1, PTGS1*	*AKR7A3, ARSG, CHST4, CHST12, CYP2D6, CYP4A11, CYP4F2, CYP4F22, CYP4X1, FMO3, UGT2A3, UGT2B10*
	BBB	*SLC3A2*	*ABCB1, ABCG2, SLC22A5, SLC25A20*
	PLC	*ADSL*	*ABCG2, HSD17B1, IL1RL1, NOTUM*
	RC	N/A	N/A
XenoReg genes with 5 or more unique predicted damaging SNVs and with no CNVs in cases (*n* = 20)	DET	*CBS, CYP1A2, NOS2*	*ALDH1L1, AOC2, AOX1, CYP2A13, CYP4F12, LOXL4*
	BBB	*AFDN, CGN, VWF*	*TJP3*
	PLC	N/A	*KIF17, NOTCH1, NOTCH3*
	RC	*CFTR, DNAH5, DNAH7, DNAH11*	N/A
XenoReg genes targeted by CNVs exclusively in cases and with no SNVs (*n* = 26)	DET	*CYP11B2, DNMT3B, GPX2, GUSB, PTGES3, STS*	*ALDH3A2, ARSE, ARSF, ARSH, CES3, CHST14, CYP4F8, CYP7A1, PTGES, SULT2B1, SUOX, UGT1A1*
	BBB	*CLDN3, SYN1*	*SLC16A1*
	PLC	N/A	*JAM2, KIFC1, TRIM64B, XAGE3*
	RC	N/A	*SGF29*
XenoReg genes with a higher proportion of CNVs in cases and with no SNVs (*n* = 7)	DET	*GSTM1*	*CYP21A2, GSTT2, UGT1A8, UGT1A10*
	BBB	N/A	N/A
	PLC	*MAGEA8*	*CSH1*
	RC	N/A	N/A

*A brief description of the function of each of these genes and data regarding previous association with ASD, when reported, is given in Supplementary Table 7. AGP, Autism Genome Project; DET, detoxification; BBB, Blood-brain barrier; PLC, placenta; RC, respiratory cilia; N/A, Not Available.*

Of the 77 high evidence XenoReg genes, 61% (47/77) encode enzymes responsible for the biotransformation of endogenous and exogenous substances, while 39% (30/77) regulate the selective permeability of physiological barriers, or play a role in their morphogenesis (Table 4 and Supplementary Table 7). Of these 77 genes, 15 (*ADSL, AFDN, CBS, CFTR, CHST5, CLDN3, CYP1A2, CYP2R1, GSTM1, GUSB, NOS2, PTGES3, STS, SYN1*, and *VWF*) have been directly implicated in ASD by previous association or expression profiling studies (Table 4 and Supplementary Table 7). Another, 8 genes (*CGN, CYP11B1, DNAH5, DNAH7, DNAH11, DNMT3A, GPX1*, and *PTGS1*) have paralogous genes cataloged in The Human Gene Module from SFARI Gene (Abrahams et al., 2013), a manually curated list of candidate genes for ASD. Additionally, the *SLC3A2* gene forms a complex with a SFARI candidate gene, and the *MAGEA8* gene is located in the Xq28 cytogenetic region previously associated with ASD. Variants in *CYP1A2* and *CYP2D6*, two pharmacogenes involved in the metabolism of psychoactive drugs used in ASD treatment, were very rare in these datasets (Table 2 and Supplementary Table 4).

For subjects with maternal genetic data available, we analyzed the transmission pattern of predicted damaging variants in high evidence genes encoding detoxification enzymes. The results revealed that, in 77/134 (57.4%) of ASC subjects, 98/210 (46.6%) of AGP subjects, and 15/33 (45.5%) of SSC subjects the variants in genes encoding detoxification enzymes were maternally-inherited (Supplementary Table 6). These results do not deviate from expected transmission rates.

### Interactions Between High Evidence XenoReg Genes and Xenobiotics Relevant for Autism Spectrum Disorder

To further explore a role for the 77 high evidence XenoReg genes in gene-environment mechanisms in ASD, we interrogated the CTD for interactions between these 77 XenoReg genes and 60 xenobiotics previously linked with ASD risk (listed in Supplementary Table 2; for additional information see Santos et al., 2021).

**FIGURE 3:**
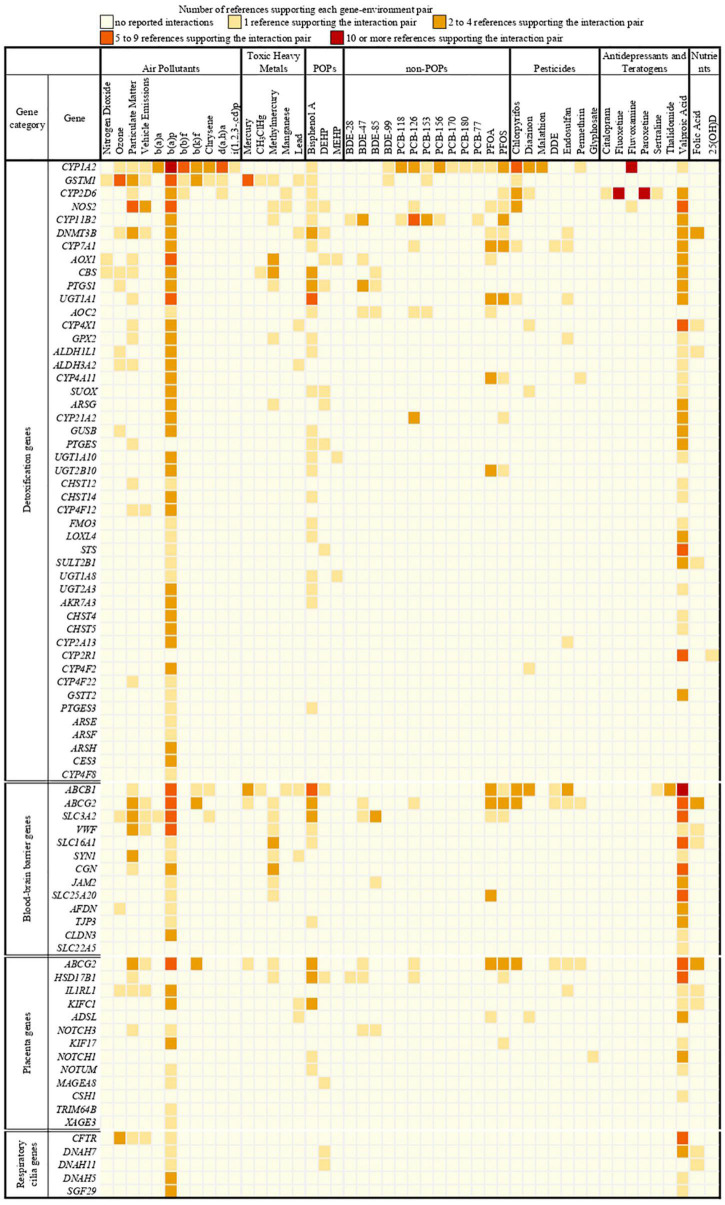
Heat map showing the gene-environment interactions pairs identified through the Comparative Toxicogenomics Database. Shown are the 397 gene-environment interaction pairs, between the 77 high evidence XenoReg genes and the 60 individual xenobiotics, identified through the CTD query. The colors represent the amount of published references supporting each interaction pair (darker colors are for high numbers of supporting references, while lighter colors are for low numbers of supporting references). Xenobiotics that do not interact with any of the 77 high evidence XenoReg genes are not shown. 25(OH)D, 25-hydroxyvitamin D; b(a)a, b(a)p, b(b)f, b(k)f, d(a,h)a and i(1,2,3,-cd)p are Polycyclic Aromatic Hydrocarbons (see Supplementary Table 2); BDE28, BDE47, BDE85, and BDE100 are congeners of Polybrominated diphenyl ethers (see Supplementary Table 2); CH_3_ClHg, Methylmercuric chloride; DDE, Dichlorodiphenyldichloroethylene; DEHP, Diethylhexyl phthalate; MEHP, Mono-(2-ethylhexyl)phthalate; non-POPs, non-Persistent Organic Pollutants; PCB118, PCB126, PCB153, PCB156, PCB170, PCB180, and PCB77 are congeners of Polychlorinated biphenyls (see Supplementary Table 2); PFOA, Perfluorooctanoic acid; PFOS, Perfluorooctane sulfonic acid; POPs, Persistent Organic Pollutants.

**FIGURE 4:**
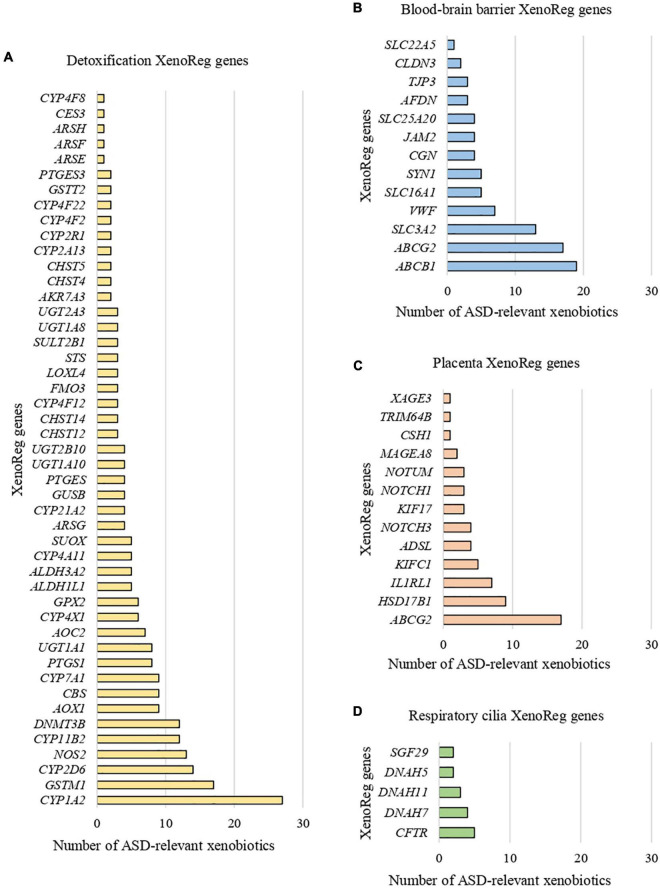
Xenobiotic interactions per high evidence XenoReg gene. Number of xenobiotics previously associated with ASD that interact with each of the 77 high evidence XenoReg genes, according to The Comparative Toxicogenomics Database. **(A)** Detoxification genes; **(B)** blood-brain barrier genes; **(C)** placenta genes; **(D)** respiratory cilia genes.

**FIGURE 5:**
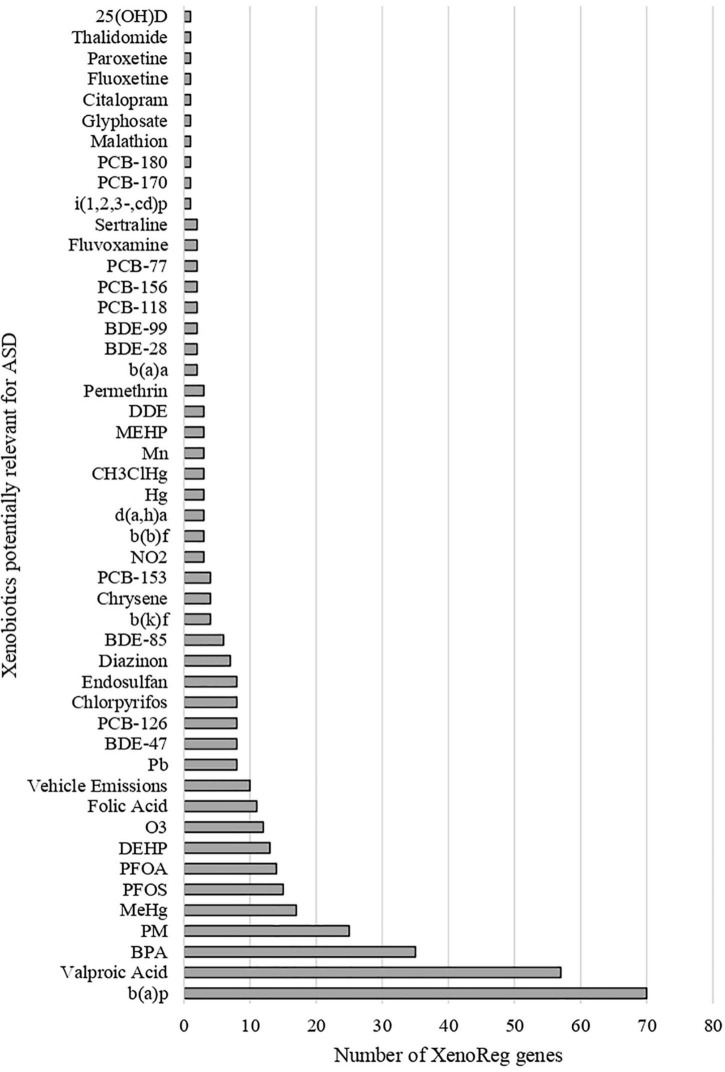
Gene interactions per xenobiotic. Number of high evidence XenoReg genes with predicted damaging variants that interact with any of the xenobiotics previously linked with ASD, according to The Comparative Toxicogenomics Database.

We identified 397 gene-environment interaction pairs, between the 77 high evidence XenoReg genes and 80% (48/60) of the xenobiotics (Figure 3). All of these 77 genes interact with at least one xenobiotic relevant for ASD, independent of whether they encode detoxification enzymes (Figure 4A), or proteins involved in permeability barrier functions (Figures 4B–D). The genes interacting with more xenobiotics were *CYP1A2* (27 chemicals), followed by two ABC transporters, *ABCB1* (19 chemicals) and *ABCG2* (17 chemicals), *GSTM1* (17 chemicals) and *CYP2D6* (14 chemicals). Most of the xenobiotics (48, 80%) interacted with one or more of these 77 genes (Figure 5). Benzo-(a)-pyrene [b(a)p] (70 genes) and valproic acid (57 genes) were the top interacting chemicals, followed by BPA (35 genes), particulate matter (PM) (25 genes), methylmercury (MeHg) (17 genes) and two PFCs, perfluorooctane sulfonic acid (PFOS) (15 genes) and perfluorooctanoic acid (PFOA) (14 genes) (Figure 5). The interaction pairs involving any of the 77 high evidence XenoReg genes are shown in the heat map in Figure 3.

## Discussion

Despite massive efforts to understand the complex genetic architecture of ASD, and the growing evidence for a role of certain xenobiotics in ASD risk, neither environmental exposure nor genetics independently have fully explained the etiology of the disorder. In the current study, we analyze large ASD genomic datasets to substantiate the hypothesis that variants in genes involved in detoxification processes or in regulating the permeability of physiological barriers to toxins may increase ASD risk upon exposure to xenobiotics.

To test this hypothesis we developed a panel of 519 key genes involved in xenobiotics detoxification or physiological permeability barriers, the XenoReg genes. This is a comprehensive panel that includes genes encoding enzymes involved in all general reactions from phase I and phase II metabolism (Ioannides, 2001; Parkinson et al., 2017). Additionally, the inclusion of genes that regulate the morphogenesis, integrity and permeability of physiological barriers allowed us to search for variants that may affect the contact of xenobiotics with the brain, particularly during neurodevelopment. According to pre-defined criteria based on *in silico* predicted functional impact and frequency of variants in the ASD population, we identified a set of 77 high evidence XenoReg genes with stronger support for an involvement in ASD.

The identification of variants predicted to be damaging *in silico* in 47 XenoReg genes encoding enzymes of phase I and II metabolic pathways is in line with previous reports of an overall impairment of detoxification processes in ASD-subjects (Alabdali et al., 2014; Bjorklund et al., 2020). The disruption of detoxification cascades can lead to the buildup of un-metabolized toxins in the bloodstream (Mitro et al., 2015), which can be especially harmful during pregnancy. The maternal-fetal unit is an interconnected system and, while toxins that enter the maternal bloodstream are metabolized by maternal enzymes, if they reach the fetus biotransformation is carried out by an immature fetal detoxification system (Barr et al., 2007). This system may be particularly inefficient in the presence of specific variants in genes encoding key enzymes. Genetic variability in these genes is known to originate variable detoxifier phenotypes, affecting the enzymatic activity and metabolizer status of variant carriers (Santos et al., 2018).

Among the high evidence detoxification genes identified with predicted damaging variants, 13 encode members of the CYP superfamily, which are major phase I enzymes responsible for the oxidative activation or deactivation of substrates. Some of these *CYPs* (e.g., *CYP1A2*, *CYP2D6*, and *CYP7A1*) encode enzymes with a very broad substrate specificity that metabolize multiple xenobiotics previously linked to ASD risk. We also identified predicted damaging variants in six members of the *CYP4* gene family (*CYP4A11*, *CYP4F12*, *CYP4F2*, *CYP4F22*, *CYP4F8*, and *CYP4X1*) which participate in the metabolism of arachidonic acid, sex steroids, and EDCs such as BPA and phthalates (Nebert et al., 2013). Conversely, *CYP2R1* specifically converts vitamin D to 25-hydroxyvitamin D, its main circulatory form. Since a growing number of studies report associations between vitamin D deficiency and ASD risk (reviewed by Mazahery et al., 2016), our findings of predicted damaging variants in the *CYP2R1* gene in ASD subjects is particularly relevant. *CYP1A2* and *CYP2D6* are important for the metabolism of psychoactive drugs, including SSRIs (Butler, 2018), and an increased hypersensitivity to these medications was described in ASD subjects compared to patients with other psychiatric disorders (Persico et al., 2021). Functional variants in *CYP1A2* and *CYP2D6* were observed in these datasets, and further investigation may elucidate on the reported hypersensitivity, improving the prediction of pharmacological safety and efficacy in carriers.

Predicted damaging variants were also found in 7 genes encoding members of two major phase II metabolism families: glutathione S-transferases (*GSTM1* and *GSTT2*) and uridine diphosphate glucuronosyltransferases (*UGT1A1*, *UGT1A8*, *UGT1A10*, *UGT2A3*, and *UGT2B10*). GSTs catalyze the conjugation of reduced glutathione to xenobiotics, while UGTs are responsible for glucuronidation reactions, in which substrates are conjugated with a glucuronic acid moiety, with the endpoint of both reactions being the increase in hydrophilicity of xenobiotics.

Among the high evidence XenoReg genes was also *STS*, which encodes steroid sulfatase, an enzyme preferentially expressed in the brain and placenta. During development, STS is involved in placental estrogen biosynthesis and catalyzes the hydrolysis of exogenous compounds, particularly those structurally similar to steroid hormones (Chatuphonprasert et al., 2018), while in the brain it maintains the balance between neurosteroids and their unconjugated forms (Kriz et al., 2008). Exposure to EDCs, such as benzo(a)pyrene and dyethylhexyl phthalate has been shown to result in an increased methylation of the *STS* 3′ UTR and in an increased expression of *STS* mRNA (Fang et al., 2019), respectively. Interestingly, we also identified predicted damaging variants in *HSD17B1*, which similarly encodes an enzyme involved in placental estrogen biosynthesis, as well as in *CYP11B2* and *CYP21A2*, which are involved in sex steroids metabolism. These genes are likely targets for dysregulation by EDCs. Many xenobiotics relevant for ASD, such as BPA and phthalates, are structurally similar to estrogens and androgens, acting as agonists and antagonists to hormone receptors (Schug et al., 2015). Given the male-to-female bias in ASD diagnoses, imbalances in sex hormones levels caused by exposure to EDCs are particularly relevant (Kern et al., 2017; Loomes et al., 2017).

Variants that alter the activity of any of these detoxification enzymes, including CYPs, GSTs, UGTs, and STS, will impact the response of the carrier to exposure to xenobiotics, and possibly explain the previously detected associations of some of these chemicals to ASD.

While variants in detoxification genes influence the degradation of xenobiotics, leading to buildup of toxins in the organism, variants in physiological barrier genes alter the integrity of the BBB, placenta and respiratory cilia, and the flow of toxins across these barriers. Some high evidence XenoReg genes with a broad substrate specificity are expressed in the BBB and/or in the placenta. For instance, the predicted damaging variants identified in the transporter genes *ABCB1*, *ABCG2*, *SLC16A1*, *SLC22A5*, *SLC25A20*, and *SLC3A2* may alter the permeability of the barriers where they are expressed to several xenobiotics (Miller, 2015; Joshi et al., 2016), increasing the risk of damaging the developing brain.

Other high evidence XenoReg genes are specifically involved in the integrity and morphogenesis of the BBB. We identified predicted damaging variants in *CGN*, *CLDN3*, *JAM2*, and *TJP3*, which encode components of the BBB tight junctions, and in *AFDN* and *SYN1*, which encode, respectively, a scaffold protein involved in tight junctions’ assembly during embryogenesis and a synapsin involved in BBB maturation. Such variants may result in a structurally deficient BBB that facilitates the traffic of toxins to the immature brain, leading to an augmented neurotoxicity that impacts downstream pathways associated with ASD, as has been suggested by previous studies (Fiorentino et al., 2016; Tarlungeanu et al., 2016). For instance, expression profiling showed an increased expression of *CLDN3* in postmortem cerebral tissues of ASD patients when compared to controls (Fiorentino et al., 2016). We also found predicted damaging variants in the gene encoding SLC3A2, a chaperone which forms a heterodimeric complex with SLC7A5. A previous study showed that the deletion of *SLC7A5* in endothelial BBB cells from mice results in decreased exploratory behavior and abnormalities in social interactions, and further identified patients with ASD carrying homozygous deletions in this gene (Tarlungeanu et al., 2016).

Some of the high evidence XenoReg genes, namely genes involved in the Notch (*NOTCH1* and *NOTCH3*) and Wnt (*NOTUM*) pleiotropic signaling pathways, are crucial for placentation (Haider et al., 2017; Robinson et al., 2017). These genes are also known to be endogenous regulators of *CYP* expression in response to exposure to xenobiotics (Thomas et al., 2015). Variants in these genes may influence placenta permeability and/or detoxification processes, and impair the signaling cascades induced by xenobiotic exposure during fetal development. We also found variants in three genes with unclear function, specifically expressed at the placenta (*MAGEA8*, *TRIM64B*, and *XAGE3*) that, according to the CTD, interact exclusively with benzo(a)pyrene or with dyethylhexyl phthalate (*MAGEA8*).

Genes encoding proteins that are crucial for the respiratory airways were also identified, including *CFTR*, which encodes an ABC transporter associated with glutathione conductance across airway epithelial cells, likely controlling local oxidative stress in response to xenobiotics exposure (Kogan et al., 2003).

Some of the high evidence XenoReg genes have previously been associated with ASD risk, supporting our results. *ADSL* and *SYN1* are cataloged as high confidence etiological genes in the SFARI Human Gene Module (category 1), while *GSTM1* is cataloged as a gene with suggestive evidence for an involvement in ASD (category 3). We also identify a number of genes (*CGN*, *CYP11B2*, *DNAH5*, *DNAH7*, *DNAH11*, *DNMT3B*, *GPX2*, and *PTGS1*) with paralogs listed in the SFARI Human Gene Module. Some high evidence XenoReg have polymorphisms and rare variants (*ADSL*, *CBS, CFTR*, *CHST5*, *CYP1A2*, *CYP2R1, GSTM1, GUSB, NOS2*, *STS*, and *SYN1*) or altered expression levels (*AFDN*, *CLDN3, PTGES3*, and *VWF*) associated with ASD (Kim et al., 2008; O’Roak et al., 2012; Braam et al., 2013; Schmidt et al., 2015; Woodbury-Smith et al., 2015; Chatterjee et al., 2016; Fiorentino et al., 2016; Patel et al., 2016; Deutsch et al., 2017; Niego and Benítez-Burraco, 2021). The predicted damaging variants uncovered in this study provide supportive evidence for their role in ASD risk, particularly in the context of early life exposure to xenobiotics.

All 77 high evidence XenoReg genes interact with at least one ASD-relevant xenobiotic, reinforcing the importance of considering the role of interactions between these genes and environmental exposure in ASD risk. Many of these genes are involved in the biotransformation of the top interacting chemicals identified: b(a)p, valproic acid, BPA, PM, MeHg, PFOA, and PFOS. B(a)p is a PAH obtained from incomplete fossil fuel combustion of carbon containing matter. It binds and activates the aryl hydrocarbon receptor which, in turn, induces the expression of genes involved in the metabolism of this PAH, including *CYP1A2, GSTM1*, and the UGT1A locus (Agrawal et al., 2018). Inducible *CYP*s convert b(a)p to genotoxic, reactive metabolites (Agrawal et al., 2018), and variants in these genes lead to an accumulation of such metabolites in the organism (Esteves et al., 2021). Valproic acid is a teratogenic drug prescribed for epilepsy and bipolar disorder, and a potent inducer of neural tube defects (Nicolini and Fahnestock, 2018). Valproic acid biotransformation involves multiple pathways, including hepatic glucuronidation by UGTs, mitochondrial β-oxidation and CYP-mediated cytosolic oxidation (Ghodke-Puranik et al., 2013). Among the genes involved in its metabolism are *UGT1A8*, *UGT1A10* (Ghodke-Puranik et al., 2013) and *SLC22A5* (Silva et al., 2008). BPA is a synthetic compound used in the production of plastics and epoxy resins, found in everyday products like food and drink packaging and toys. As a non-POP, it is rapidly excreted by the organism, but BPA exposure is widespread among the general population. In humans, the main route of BPA metabolism is carried out by UGTs, including *UGT1A1*, with sulfate conjugation by sulfotransferases being also important (Kang et al., 2006; Street et al., 2017). PM is a mixture of solid particles and liquid droplets found in the atmosphere. Given its heterogeneous composition there is no single metabolic pathway for PM, but phase I and II enzymes, including CYPs and GSTs, are involved in the degradation of its components (Kelly and Fussell, 2012). MeHg is a toxic compound formed in aquatic systems and, because it is not readily biodegraded, it accumulates up the food chain, with fish consumption being the major source of exposure in humans. The pathways involved in MeHg metabolism are poorly defined, but it is known that *GSTM1* is necessary for conjugation with glutathione (Llop et al., 2017). In this study we did not consider etHg, since a role of thimerosal exposure and vaccination in ASD has been discredited (Taylor et al., 2014). etHg also has a limited capacity of crossing the BBB due to its hydrophilic structure, and a much lower half-life when compared to MeHg, not building up toxic levels in the body (Hurley et al., 2010). PFOA and PFOS are POPs used in industrial and commercial settings that cannot be metabolized in mammals, with excretion being the only means by which these compounds are eliminated (Stahl et al., 2011). However, one study found that in human liver cells there was a reduction in the expression of the high evidence XenoReg genes *CYP1A2* and *UGT1A1* upon exposure to PFOA or PFOS and upon exposure to only PFOA, respectively (Franco et al., 2020). This suggests that exposure to PFCs dysregulates biotransformation pathways, which is important in carriers of predicted damaging variants in XenoReg genes.

According to the CTD, other relevant xenobiotics including pesticides, phthalates and PBDEs interact with fewer XenoReg genes. This might reflect a gap in the study of these chemicals, as experimentally assessing the effects of thousands of existing chemicals is a difficult task (Landrigan, 2010). Interactions between multiple xenobiotics may also have additive, synergistic or antagonistic effects (Schug et al., 2015), but little data exists on how exposure to chemical mixtures may impact neurodevelopment. To fully comprehend the role of gene-environment in ASD, future studies need to address exposure to multiple xenobiotics, adopting an exposomics approach.

Early life exposure to the xenobiotics previously implicated in ASD is known to induce neuropathological processes like epigenetic alterations, oxidative stress, neuroinflammation, hypoxic damage, and endocrine disruption (Modabbernia et al., 2017; Cheroni et al., 2020). Such processes have been repeatedly associated with ASD. For instance, exposure to valproic acid (Phiel et al., 2001; Grafodatskaya et al., 2010), BPA (Kundakovic et al., 2013; Alavian-Ghavanini et al., 2018), PM (Zhao et al., 2021), and MeHg (Culbreth and Aschner, 2019) is known to alter epigenetic patterns of genes associated with ASD, such as *MECP2*, *GRIN2B*, *DNMT3A*, and *ESR2*. Biomarkers of elevated oxidative stress (Waligóra et al., 2019), fetal hypoxic damage (Burstyn et al., 2011), and neuroinflammation (Eissa et al., 2020) are also commonly observed in ASD patients. These mechanisms are elicited as a response to cumulative exposure to xenobiotics, which can be caused by deficient detoxification mechanisms or ineffective physiological barriers. Overall, our results suggest that subjects carrying predicted damaging variants in XenoReg genes may accumulate un-metabolized xenobiotics or their toxic metabolites, due to an impaired detoxification system and/or a compromised permeability of physiological barriers. These toxic substances ultimately reach the immature brain and dysregulate neuronal processes known to be implicated with ASD.

In the current work we applied a population-based approach, with *in silico* variant analysis and database exploration, to gather evidence for our hypothesis that gene-environment interactions play an important role is ASD. However, we did not have access to individualized data regarding early life environmental exposures for the studied population. Having access to personal exposure data would allow us to understand if a given variant could have modulated the response of the carrier to the exposure to a specific xenobiotic. Future efforts should be made toward the collection of genetic, exposure and clinical data from the same individuals with ASD, and the experimental *in vivo* analysis of interactions between XenoReg genes and xenobiotics.

Common variants play an important role in ASD (Grove et al., 2019), and functional polymorphisms in biotransformation enzymes such as *PON1* (D’Amelio et al., 2005), *GSTM1* (Bach et al., 2020), and *GSTP1* (Rahbar et al., 2018) have been suggested to mediate ASD risk through interactions with environmental exposure. In this study we focused on rare variants and did not analyze those with a MAF > 5% in the general population. The discovery of common variants could be relevant in future studies for the estimation of polygenic risk scores. Future studies will also benefit from understanding whether interactions between multiple variants present in the same subject may act additively to increase his or her susceptibility to early life exposures. Similarly, exploring interactions of xenobiotics with other genes, including known candidates for ASD, may provide a more complete understanding of the mechanisms underlying gene-environment interactions in the disorder (Henriksen et al., 2020).

During pregnancy, maternal detoxification enzymes constitute the first line of defense against environmental exposure. Because maternal variants in genes encoding detoxification enzymes influence the quantity and the status (more or less hydrophilic, activated, or inactivated) of the xenobiotics that reach the fetus, it is important to characterize their transmission pattern. This could help identify individuals with an increased risk of ASD due to environmental exposure, because their mothers could already have a compromised detoxification system. Our analysis showed that predicted damaging variants in detoxification genes were inherited from their mothers in half of the subjects, as expected. This analysis was incomplete due to missing genetic data, particularly for the ASC dataset.

Overall, future research can leverage from integrating information on polymorphisms and gene-gene interactions with environmental data, as well as assessing the transmission pattern of detected variants, to better comprehend the multifactorial landscape of ASD.

From this and previous studies, an involvement of both genetic and environmental risk factors in ASD etiology is clearly emerging. Pioneering research programs, such as the Childhood Autism Risks from Genetics and Environment (CHARGE) (Hertz-Picciotto et al., 2006), the Center for the Health Assessment of Mothers and Children of Salinas (CHAMACOS) (Eskenazi et al., 2003) and the Markers of Autism Risk in Babies—Learning Early Signs (MARBLES) (Hertz-Picciotto et al., 2018) are providing valuable knowledge on the contribution of environmental factors to ASD, while also shedding light on selected genes. However, comprehensive research addressing gene-environment interactions is still lacking. The present study contributes to fill this gap, by providing evidence that subjects with ASD carry damaging variants in a large set of genes involved in detoxification or regulation of physiological barriers to xenobiotics previously associated with the disorder.

## Conclusion

The results of the multilevel strategy adopted in this study support the hypothesis that XenoReg genes, involved in detoxification mechanisms or the function of physiological barriers, can impact ASD risk by regulating the effects of xenobiotic exposure. Using large genomic datasets, we found that subjects with ASD carry predicted damaging variants in prioritized XenoReg genes with diverse functions, including some already reported candidates for the disorder. By querying the CTD, a public, manually curated database, we also show that these genes interact with xenobiotics previously implicated in ASD. We describe known neuropathological processes that can be triggered by xenobiotic exposure in carriers of risk variants, who will have difficulties in detoxifying or filtering out specific xenobiotics with possible consequences for neurodevelopment. In the absence of individualized exposure data, this strategy led to findings that can be leveraged to guide future work, for instance to select relevant gene-xenobiotic interaction pairs for further investigation.

ASD is an extremely complex disorder, with a very heterogeneous clinical presentation, and unexplained etiology for a large number of cases. Even though there is growing evidence implicating environmental factors in ASD, the lack of detailed exposure data in large ASD population datasets is hindering our understanding of the impact of interactions between genetic profiles and environmental exposure. This is, however, a very enticing hypothesis because the diversity of interactions can match, and possibly explain, the phenotypic heterogeneity that characterizes this disorder. Most importantly, exposure to environmental factors may be mitigated for individuals with XenoReg gene risk variants, and therefore understanding gene-environment interactions opens the perspective for personalized prevention and effective health management policies for ASD.

## Data Availability Statement

Publicly available datasets were analyzed in this study. Data from the Genome Aggregation Database (RRID: SCR_014964), the Database of Genomic Variants (RRID: SCR_007000), and the Comparative Toxicogenomics Database (RRID: SCR_006530) are publicly available. Data from Autism Sequencing Consortium and Autism Genome Project can be found in the database of Genotypes and Phenotypes (dbGaP) repository, with the accession codes phs000298.v4.p3 and phs000267.v5.p2, respectively.

## Ethics Statement

The studies involving human participants were reviewed and approved by Comissão de Ética para a Saúde do Instituto Nacional de Saúde Doutor Ricardo Jorge, I.P. (INSA, I.P.) and by Comissão de Ética do Centro Hospitalar e Universitário de Coimbra (CE-CHUC). Written informed consent to participate in this study was provided by the participants’ legal guardian/next of kin.

## Author Contributions

AMV, JXS, and CR developed the concept for this work. JXS carried out data curation, methodological development, and formal analysis, with strong support from ARM, HM, and MA and with supervision from LS and AMV. GO and AMV provided resources, including funding. AMV and AN performed the overall supervision and coordination. JXS wrote the initial draft, revised and edited by AMV and reviewed by CR, HM, MA, JV, GO, LS, and AN. All authors contributed to the article and approved the submitted version.

## Conflict of Interest

The authors declare that the research was conducted in the absence of any commercial or financial relationships that could be construed as a potential conflict of interest.

## Publisher’s Note

All claims expressed in this article are solely those of the authors and do not necessarily represent those of their affiliated organizations, or those of the publisher, the editors and the reviewers. Any product that may be evaluated in this article, or claim that may be made by its manufacturer, is not guaranteed or endorsed by the publisher.

## Footnotes

1
http://www.proteinatlas.org
