# Supplementary material for: A Role for Gene-Environment Interactions in Autism Spectrum Disorder Is Supported by Variants in Genes Regulating the Effects of Exposure to Xenobiotics
Source: Front Neurosci. 2022 May 19;16:862315. doi: 10.3389/fnins.2022.862315 (PMC9161282; doi:10.3389/fnins.2022.862315)
Supplement: Supplementary file 1 [file Table_1.docx]

Supplementary Material

**Supplementary Table 1.** Data regarding ASD and control population datasets used in this study. *ASC – Autism Sequencing Consortium; ASD – Autism Spectrum Disorder; AGP – Autism Genome Project; SSC – Simons Simplex Collection*

| **Dataset** | **Population type** | **Number of analyzed subjects** | **Ethnicity (%)** | **References** |
| --- | --- | --- | --- | --- |
| ASC | ASD-families and unrelated controls | 2,674 ASD subjects and 752 controls | Caucasians (100%) | Buxbaum et al., 2012 |
| AGP | Simplex and multiplex families | 2,446 subjects with ASD | Caucasians (87.8%); African-Americans (3.1%); Latin-Americans (6.3%); Asians (2.8%); Mixed (0.1%) | Hu-Lince et al., 2005; Pinto et al., 2010, 2014 |
| SSC | Simplex families | 1,124 subjects with ASD | Caucasians (78.6%); African-Americans (3.8%); Latin-Americans (4%); Asians (4.3%); Mixed (9.3%) | Fischbach and Lord 2010; Sanders et al., 2011 |
| Cooper et al., 2011 control cohort | Unrelated subjects | 8,329 subjects without history of neuropsychiatric disease | Caucasians (81.2%); African-Americans (2%); individuals of other or mixed ancestry (16.5%). | Cooper et al., 2011 |
| Shaikh et al., 2009 control cohort | Unrelated subjects | 1,320 subjects without history of neuropsychiatric disease | Caucasians (100%) | Shaikh et al., 2009 |

**Supplementary Table 2.** Xenobiotics potentially relevant for ASD studied for interactions with XenoReg genes. Individual xenobiotics potentially relevant for ASD studied for interactions with genes involved in detoxification and regulation of barriers permeability, using the Comparative Toxicogenomics Database. Multiple PAHs, PBDEs, PCBs, PFCs and phthalate congeners were analyzed. Compounds containing mercury (*i.e.* CH_3_ClHg and MeHg) were included in the heavy metals. The five SSRIs included are the ones most commonly marketed for pharmaceutical purposes. Circulatory levels of 25(OH)D, the precursor of the active form of vitamin D, are usually quantified to assess vitamin D deficiency, thus the inclusion as a nutritional factor.

| **Category** | **Individual chemical** | **MeSH ID** | **Category** | **Individual chemical** | **MeSH ID** |
| --- | --- | --- | --- | --- | --- |
| **Air Pollutants** | Nitrogen dioxide (NO_2_) | D009585 | **Persistent Organic Pollutants** | **Polybrominated diphenyl ethers (PBDEs)** | |
|  | Ozone (O_3_) | D010126 |  | 2,2',3,4,4'-pentabromodiphenyl ether (BDE-85) | C086401 |
|  | Particulate matter (PM) | D052638 |  | 2,2',4,4',5-brominated diphenyl ether (BDE-99) | C477694 |
|  | Sulfur Dioxide (SO_2_) | D013458 |  | 2,2',4,4',6-brominated diphenyl ether (BDE-100) | C517827 |
|  | Vehicle emissions | D001335 |  | 2,2',4,4'-tetrabromodiphenyl ether (BDE-47) | C511295 |
|  | **Polycyclic Aromatic Hydrocarbons (PAHs)** | |  | 2,2',4-tribromodiphenyl ether (BDE-28) | C533760 |
|  | Benzo(a)anthracene [b(a)a] | C030935 |  | **Polychlorinated biphenyls (PCBs)** | |
|  | Benzo(a)pyrene [b(a)p] | D001564 |  | 2,2',3,3',4,4',5-heptachlorobiphenyl (PCB-170) | C541131 |
|  | Benzo(b)fluoranthene [b(b)f] | C006703 |  | 2,2',3,4,4',5,5'-heptachlorobiphenyl (PCB-180) | C410127 |
|  | Benzo(g,h,i)perylene [b(g,h,i)p] | C006718 |  | 2,2',3',4,4',5-hexachlorobiphenyl (PCB-138) | C029790 |
|  | Benzo(k)fluoranthene [b(k)f] | C022921 |  | 2,3,3',4,4',5-hexachlorobiphenyl (PCB-156) | C087667 |
|  | Chrysene | C031180 |  | 2,3',4,4',5-pentachlorobiphenyl (PCB-118) | C070055 |
|  | Dibenzo(a,h)anthracene [d(a,h)a] | C026486 |  | 2,4,5,2',4',5'-hexachlorobiphenyl (PCB-153) | C014024 |
|  | Indeno(1,2,3,-cd)pyrene [i(1,2,3,-cd)p] | C041508 |  | 3,4,3',4'-tetrachlorobiphenyl (PCB-77) | C028451 |
| **Non-Persistent Organic Pollutants** | Bisphenol A | C006780 |  | 3,4,5,3',4'-pentachlorobiphenyl (PCB-126) | C023035 |
|  | **Phthalates** | |  | **Perfluorinated compounds (PFCs)** | |
|  | Butylbenzyl phthalate (BBzP) | C027561 |  | Perfluorooctane sulfonic acid (PFOS) | C076994 |
|  | Dibutyl phthalate (DBP) | D003993 |  | Perfluorooctanoic acid (PFOA) | C023036 |
|  | Diethyl phthalate (DEP) | C007379 | **Pesticides** | **Organochlorine Pesticides** | |
|  | Diethylhexyl phthalate (DEHP) | D004051 |  | Dichlorodiphenyldichloroethylene (DDE) | D003633 |
|  | Dimethyl phthalate (DMP) | C024629 |  | Dicofol | D004010 |
|  | Mono-(2-ethylhexyl)phthalate (MEHP) | C016599 |  | Endosulfan | D004726 |
| **Heavy Metals** | Lead (Pb) | D007854 |  | **Organophosphate Pesticides** | |
|  | Manganese (Mn) | D008345 |  | Chlorpyrifos | D004390 |
|  | Mercury (Hg) | D008628 |  | Diazinon | D003976 |
|  | Methylmercuric chloride (CH_3_ClHg) | C004925 |  | Dimethylthiophosphate (DMTP) | C040340 |
|  | Methylmercury compounds (MeHg) | D008767 |  | Malathion | D008294 |
| **Clinical drugs** | **Selective serotonin reuptake inhibitors** | |  | **Pyrethroids** | |
|  | Citalopram | D015283 |  | Permethrin | D026023 |
|  | Fluoxetine | D005473 |  | **Other pesticides** | |
|  | Fluvoxamine | D016666 |  | Glyphosate | C010974 |
|  | Paroxetine | D017374 | **Nutritional Factors** | Folic acid | D005492 |
|  | Sertraline | D020280 |  | 25-hydroxyvitamin D [25(OH)D] | C104450 |
|  | **Teratogenic medications** | |  | Vitamin D | D014807 |
|  | Thalidomide | D013792 |  |  |  |
|  | Valproate (VPA) | D014635 |  |  |  |
|  | Misoprostol | D016595 |  |  |  |

**References (for Supplementary Table 1 and Supplementary Table 7):**

1. Alex AM, Saradalekshmi KR, Shilen N, Suresh PA, Banerjee M. (2019). Genetic association with DNMT variants can play a critical role in defining the methylation patterns in autism. *IUBMB Life* 71(7):901-907. doi: 10.1002/iub.2021.
2. Belardo A, Gevi F, Zolla L. (2019). The concomitante lower concentrations of vitamins B6, B9, B12 may cause methylation deficiency in autistic children. *J Nutr Biochem.* 70_38-46. doi: 10.1016/j.jnutbio.2019.04.004
3. Bjorklund G, Tinkov AA, Hosnedlová B, Kizek R, Ajsuvakova OP, Chirumbolo S, et al. (2020). The role of glutathione redox imbalance in autism spectrum disorder: A review. *Free Radic Biol Med.* 160:149-162. doi: 10.1016/j.freeradbiomed.2020.07.017
4. Braam W, Keijzer H, Boudier HS, Didden R, Smits M, Curfs L, et al. (2013). CYP1A2 polymorphisms in slow melatonin metabolisers: a possible relationship with autism spectrum disorder? *J Intellect Disabil Res.* doi: 10.1111/j.1365-2788.2012.01595.x
5. Brandler WM, Antaki D, Gujral M, Noor A, Rosanio G, Chapman TR, et al. (2016). Frequency and complexity of de novo structural mutation in autism. *Am J Hum Genet.* 98(4):667-679. doi:10.1016/j.ajhg.2016.02.018
6. Buxbaum JD, Daly MJ, Devlin B, Lehner T, Roeder K, State MW, Autism Sequencing Consortium. (2012). The Autism Sequencing Consortium: Large-Scale, High-Throughput Sequencing in Autism Spectrum Disorders. *Neuron* 76(6):1052–6. doi: 10.1016/j.neuron.2012.12.008
7. Celestino-Soper PBS, Shaw CA, Sanders SJ, Li J, Murtha MT, Ercan-Sencicek AG, et al. (2011). Use of array CGH to detect exonic copy number variants throughout the genome in autism families detects a novel deletion in TMLHE. *Hum Mol Genet.* 20(22):4360-4370. doi: 10.1093/hmg/ddr363
8. Chatterjee S, Humby T, Davis W. (2016). Behavioral and psychiatric phenotypes in men and boys with X-linked ichthyosis: evidence from a worldwide online survey. *PLoS One* 11(10):e0164417. doi 10.1371/journal.pone.0164417
9. Chatuphonprasert W, Jarukamjorn K, Ellinger I. (2018). Physiology and pathophysiology of steroid biosynthesis, transport and metabolism in the human placenta. *Front Pharmacol.* 9:1027. doi: 10.3389/fphar.2018.01027
10. Cooper GM, Coe BP, Girirajan S, Rosenfeld JA, Vu TH, Baker C, et al. (2011). A copy number variation morbidity map of developmental delay. *Nat Genet.* 43(9):838–46. doi: 10.1038/ng.909
11. Cukier HN, Dueker ND, Slifer SH, Lee JM, Whitehead PL, Lalanne E, et al. (2014). Exome sequencing of extended families with autism revels genes shared across neurodevelopmental and neuropsychiatric disorders. *Mol Autism* 5(1):1. doi: 10.1186/2040-2392-5-1.
12. Deutsch SI, Kreiser NL, Urbano MR, Burker JA, Picket JC. (2017). Autism presenting in the context of a genetic variant of *CFTR* and early HSV exposure confounded by chronic pain, altered gut microbiota and paternal abandonment: limitations of current pharmacotherapy and barriers to personalized treatment recommendations. *Personal Med Psychiatry* 3:24-29. doi.org/10.1016/j.pmip.2017.07.002
13. El Shafae M, Sabry JH, Behiry EG, Elshahat SA, Zaki MS, Esmaiel NN. (2017). Association of cystathionine β synthase gene polymorphism with cognitive disorders in autistic children. *JIPBS* 4, 5.
14. Engchuan W, Dhindsa K, Lionel AC, Scherer SW, Chan JH, Merico D. (2015). Performance of case-control rare copy number variation annotation in classification of autism. *BMC Med Genomics* 8 Suppl 1(Suppl 1):S7. doi: 10.1186/1755-8794-8-S1-S7.
15. Fassio A, Patry L, Congia S, Onofri F, Piton A, Gauthier J, et al. (2011). SYN1 loss-of-function mutations in autism and partial epilepsy cause impaired synaptic function. *Hum Mol Genet* 20(12):2297-2307. doi: 10.1093/hmg/ddr122.
16. Fiorentino M, Sapone A, Senger S, Camhi SS, Kadzielski SM, Buie TM, et al. (2016). Blood-brain barrier and intestinal epithelial barrier alterations in autism spectrum disorders. *Mol Autism* 7:49. doi: 10.1186/s13229-016-0110-z
17. Fischbach GD, Lord C. (2010). The Simons Simplex Collection: a resource for identification of autism genetic risk factors. *Neuron* 68(2):192-5. doi: 10.1016/j.neuron.2010.10.006
18. Glatt SJ, Tsuang MT, Winn M, Chandler SD, Collins M, Lopez L, et al. (2013). Blood-based gene expression signatures of autistic infants and toddlers. *J Am Acad Child Adolesc Psychiatry* 51(9):934-944. doi: 10.1016/j.jaac.2012.07.007
19. Guillemot L, Schneider Y, Brun O, Castagliuolo, Pizzuti D, Martines D, et al. (2012). Cingulin is dispensable for epithelial barrier function and tight junction structure, and plays a role in the control of claudin-2 expression and response to duodenal mucosa injury. *J Cell Sci.* 125(Pt 21):5005-5014. doi: 10.1242/jcs.101261.
20. Haider S, Pollheimer J, Knolfer M. (2017). Notch signalling in placental development and gestational diseases. *Placenta* 56:65-762. doi: 10.1016/j.placenta.2017.01.117
21. Hu-Lince D, Craig DW, Huentelman MJ, Stephan DA. (2005). The Autism Genome Project: goals and strategies. *Am J Pharmacogenomics* 5(4):233-46. doi: 10.2165/00129785-200505040-00004
22. James SJ, Cutler P, Melnyk S, Jernigan S, Janak L, Gaylor DW, Neubrander JA. (2004). Metabolic biomarkers of increased oxidative stress and impaired methylation capacity in children with autism. *AM J Clin Nutr.* 80(6):1611-1617. doi: 10.1093/ajcn/80.6.1611
23. Kent L, Emerton J, Bhadravathi V, Weisblatt E, Pasco G, Willatt LR, et al. (2008). X-linked ichthyosis (steroid sulfatase deficiency) is associated with increased risk of attention deficit hyperactivity disorder, autism and social communication deficits. *J Med Genet.* 45(8):519-524. doi: 10.1136/jmg.2008.057729.
24. Kharkwal H, Batool F, Koentgen F, Bell DR, Ebling FJP, Duce IR. (2017). Generation of phenotypic characterization of a cytochrome P450 4x1 knockout mouse. *PLoS One* 12(12):e0187959. doi: 10.1371/journal.pone.0187959
25. Kriz L, Bicikova M, Hampl R. (2008). Roles of steroid sulfatase in brain and other tissues. *Physiol Res.* 57(5):657-668. doi: 10.33549/physiolres.931207
26. Krumm N, Turner TN, Baker C, Vives L, Mohajeri K, Witherspoon K, et al. (2015). Excess of rare, inherited truncating mutations in autism. *Nat Genet.* 47(6):582-588. doi: 10.1038/ng.3303.
27. Melrose J. (2019). Keratan sulfate (KS)-proteoglycans and neuronal regulation in health and disease: the importance of KS-glycodynamics and interactive capability with neuroregulatory ligands. *J Neurochem* 149(2):180-194. doi: 10.1111/jnc.14652
28. Miller B, Sheppard AM, Pearlman AM. (1997). Developmental expression of keratin sulfate-like immunoreactivity distinguishes thalamic nuclei and cortical domains. *J Comp Neurol.* 380(4):533-552. doi: 10.1002/(sici)1096-9861(19970421)380:4<533::aid-cne9>3.0.co;2-2.
29. Modabbernia A, Velthorst E, Reichenberg A. (2017). Environmental risk factors for autism: an evidence-based review of systematic reviews and meta-analyses. *Mol Autism* 8(1):1–16. doi: 10.1186/s13229-017-0121-4
30. Niego A, Benítez-Burraco A. (2021). Autism and Williams syndrome: Dissimilar socio-cognitive profiles with similar patterns of abnormal gene expression in the blood. *Autism* 25(2):464-489. doi: 10.1177/1362361320965074
31. O’Roak BJ, Vives L, Girirajan S, Karakoc E, Krumm N, Coe BP, et al. (2012). Sporadic autism exomes reveal a highly interconnected protein network of de novo mutations. *Nature* 485(7397):246-50. doi: 10.1038/nature10989.
32. Ozato K, Shin DM, Chang TH, Morse HC. (2008). TRIM family proteins and their emerging roles in innate immunity. *Nat Rev Immunol.* 8(11):849-860. doi: 10.1038/nri2413.
33. Patel N, Crider A, Pandya CD, Ahmed AO, Pillai A. (2016). Altered mRNA levels of glucocorticoid receptor, mineralocorticoid receptor, and co-chaperones (FKBP5 and PTGES3) in the middle frontal gyrus of autism spectrum disorder subjects. *Mol Neurobiol.* 53(4): 2090-2099. doi: 10.1007/s12035-015-9178-2
34. Pinto D, Pagnamenta AT, Klei L, Anney R, Merico D, Regan R, et al. (2010). Functional Impact of Global Rare Copy Number Variation in Autism Spectrum Disorder. *Nature* 466(7304):368–72. doi: 10.1038/nature09146
35. Pinto D, Delaby E, Merico D, Barbosa M, Merikangas A, Klei L, et al. (2014). Convergence of Genes and Cellular Pathways Dysregulated in Autism Spectrum Disorders. *Am J Hum Genet.* 94(5):677–94. doi: 10.1016/j.ajhg.2014.03.018
36. Robinson JF, Kapidzic M, Gormley M, Ona K, Dent T, Seifikar H, et al. (2017). Transcriptional dynamics of cultured human villous cytotrophoblasts. *Endocrinology* 158(6):1581-1594. doi: 10.1210/en.2016-1635
37. Sanders SJ, Ercan-Sencicek AG, Hus V, Luo R, Murtha MT, Moreno-de-Luca D, et al. (2011). Multiple recurrent de novo CNVs, including duplications of the 7q1111.23 Williams syndrome region, are strongly associated with autism. *Neuron* 70(5):863-85. doi: 10.1016/j.neuron.2011.05.002
38. Schmidt RJ, Handen RL, Hartiala J, Allayee H, Sconberg JL, Schmidt LC, et al. (2015). Selected vitamin D metabolic gene variants and risk for autism spectrum disorder in the CHARGE study. *Early Hum Dev.* 91(8): 483–489. doi: 10.1016/j.earlhumdev.2015.05.008
39. Shaikh T, Gai X, Perin JC, Glessner JT, Xie H, Murphy K, O'Hara R et al. (2009). High-resolution mapping and analysis of copy number variations in the human genome: A data resource for clinical and research applications. *Genome Res.* 19(9)1682–90. doi: 10.1101/gr.083501.108
40. Sivendran S, Patterson D, Spiegel E, McGown I, Cowley D, Colman RF. (2004). Two novel mutant human adenolysuccinate lyases (ASLs) associated with autism and characterization of the equivalent mutant Bacillus subtilis ASL. *J Biol Chem.* 279(51):53789-97. doi: 10.1074/jbc.M409974200
41. Tarlungeanu DC, Deliu E, Dotter CP, Kara M, Janiesch PC, Scalise M, et al. (2016). Impaired amino acid transport at the blood brain barrier is a cause of autism spectrum disorder. *Cell* 167(6):1481-94. doi: 10.1016/j.cell.2016.11.013
42. Veatch OJ, Pendergast J, Allen MJ, Leu RM, Johnson CH, Elsea SH, et al. (2015). Genetic variation in melatonin pathway enzymes in children with autism spectrum disorder and comorbid sleep onset delay. *J Autism Dev Disord.* 45(1):100-110. doi: 10.1007/s10803-014-2197-4.
43. Wang S, Cesca F, Loers G, Schweizer M, Buck F, Benfenati F, et al. (2011). Synapsin I is a oligomannose-carrying glycoprotein, acts as an oligamannose-binding lectin, and promotes neurite outgrowth and neuronal survival when released via glia-derived exosomes. *J Neurosci.* 31(20):7275-7290. doi: 10.1523/JNEUROSCI.6476-10.2011.
44. Woodbury-Smith M, Paterson AD, Thiruvahindrapduram B, Lionel AC, Marshall CR, Merico D, et al. (2014). Using extended pedigrees to identify novel autism spectrum disorder (ASD) candidate genes. *Hum Genet.* 134(2):191-201. doi: 10.1007/s00439-014-1513-6
45. Wong-Riley MTT and Besharse JC. (2012). The kinesin superfamily protein KIF17: one protein with many functions. *Biomol Concepts* 3(3):267-282. doi: 10.1515/bmc-2011-0064.
